# Characteristics of surface modified sugarcane bagasse cellulose: application of esterification and oxidation reactions

**DOI:** 10.1038/s41598-024-75846-8

**Published:** 2024-10-15

**Authors:** Sithara Rao, M. Madhushree, K Subrahmanya Bhat

**Affiliations:** Department of Chemistry, Manipal Institute of Technology, Manipal Academy of Higher Education, 576104 Manipal, Karnataka India

**Keywords:** Bagasse fibers, Chemical modifications, Cellulose fibers, Esterification, Oxidation, Characterizations, Chemistry, Materials science

## Abstract

Research on polymer matrix composites has become increasingly important in both the academic and industrial sectors. The study of polymer-natural fiber composites, known for their eco-friendly properties, has gained significance. Sugarcane bagasse fibers, abundant as discarded agricultural byproducts, offer improved properties such as density, rigidity, strength, and cost-effectiveness, enhancing sustainability. As a result, experiments were performed on cellulose fibers pre-treated from sugarcane bagasse using 5% NaOH solution by simply soaking them for 4–5 h followed by washing with water. Further modifications involved esterification using phthalic anhydride and phthaloyl chloride via steam baths at 90 °C and oxidation using sodium percarbonate with a phase transfer catalyst (Adogen) at 80 °C. These chemically altered cellulose fibers exhibited significant peak changes in the FTIR spectra, a reduced crystallinity index in the XRD pattern, increased thermal stability as evidenced by TGA curve, and improved surface roughness in the SEM analysis. This paper emphasizes successful pretreatment procedures for isolating cellulose fibers from sugarcane bagasse and introduces three chemical treatments for surface functionalization which might find applications in the preparation of biocomposites.

## Introduction

Polymers and polymer composites have attracted renewed interest worldwide for their variety of applications. The simplicity of processing, light weight, increased production, and cost savings are the most crucial characteristics making them valuable in the manufacture of tools and parts of devises. Several of these applications require a high strength and modulus; hence the polymer characteristics are enhanced by utilizing fillers and fibers. One area of research has been the use of natural fibers derived from plants as reinforcement agents in polymer composites which may be more affordable, durable, and eco-friendly^1,2^.

Polymer matrix composites (PMCs) can improve properties such as fracture toughness, strength, and stiffness by integrating organic polymers with short or continuous fibers and reinforcing agents. The purpose of the matrix is ​​to retain the fibers together so that loads can be effectively transferred between them. The fibers are the main building blocks of the load carriers, and the matrix binds them in place while the correct arrangement transfers the load between them protecting them from environmental damage^1,3^.

With a few extractives, these fibers mostly comprise cellulose, hemicelluloses, lignin, and pectin. Natural fibers provide various benefits over glass and carbon fibers, including increased availability due to potential for cultivation, affordability, biodegradability, easy processing, reduced machine wear, limited health risks, low density, an ideal fiber aspect ratio, and a comparatively high tensile modulus^4–6^.

The characteristics of cellulosic fibers are influenced primarily by the region in which they are cultivated, the type of plant, the method of extraction, and the age of the plant. For instance, multicellular fibers such as coir and oil palm fibers have a core fraction known as the lacuna that is tough. In contrast, the high cellulose content of sisal and pineapple leaf fibers makes them extraordinarily tough and flexible^7^.

Sugarcane bagasse (SCB) contains 40–50% cellulose, much of which is basically crystalline. Other constituents like hemicelluloses, amorphous polymers often made of xylose, arabinose, galactose, glucose, and mannose, make up the remaining 25–35%. Other material is lignin, along with smaller amounts of substances including wax and minerals. Chemically, cellulose is a linear natural polymer made up of anhydrous-glucose units joined by *β*-glycosidic bonds at the one and four carbon atom position of the glucose unit^8^. The chemical components (%, w/w) of the SCB were 43.6% cellulose, 33.5% hemicellulose, 18.1% lignin, 2.3% ash, and 0.8% wax on a dry weight basis^9–12^.

Hemicellulose, which has a branching structure and a lower molecular weight than cellulose is composed of 6- and 5-carbon ring sugars. After the lignin component has been removed, hemicellulose remains linked to the cellulose^13^. Lignin is the most heavily branched polymer of all the components that make up the fibril wall unit. The specific cells of tough fibers serve as cementing agents by holding together and binding together other fibers. The characteristics, structure, and morphology of plant-based fibers are also influenced by the lignin content^7^.

Hemicellulose has a degree of polymerization of 30–50 and is soluble in both alkaline and acidic solutions while lignin remains unstructured, mostly hydrophobic, and insoluble in many of the solvents. The final part of the fiber is wax, which is made up of many types of alcohol. Differences in the composition, structure, and properties of each type of fiber depend on factors such as growth, climatic conditions, and the extraction approach used^13^.

However, many limitations prevent the use of natural fibers as reinforcements in polymers, including the incompatibility between fibers and polymer matrices, the ability to agglomerate during manufacture, and low moisture resistance. To enhance the fiber interface, several structural modifications and chemical coupling agents are considered^14^.

Bacteria and fungi are examples of microscopic creatures that can be used to biologically treat fibers. These methods are slow but inexpensive and eco-friendly. Peroxidases and other enzymes are employed for enhancing the functionality of lignocellulose, e.g., the enzyme laccase was used to treat fibers which resulted in a significant reduction in lignin^10^. To increase the surface tension, morphology, chemical composition, and functional characteristics of plant fibers, various physical treatments, including plasma, corona, gamma, and ultraviolet radiation, can be used^12,15^.

Chemical alterations to fiber surfaces including acetylation, mercerization, methylation, cyanoethylation, benzoylation, permanganate treatment, acrylation, and esterification can decrease the amount of moisture that fibers absorb^11,16^. Natural fibers after chemical treatments reported to improve their exterior shape, remove impurities, increase fibril strength, and prevent interactions between the fibril and the matrix. Such treatments remove surface impurities which improves wetting between the polymers and fiber possibly due to the formation of additional covalent bonds, and or by developing s a cross-linked structure at interphase region^17,18^.

The benefits of chemical treatment include simplicity in processing, versatility in application and execution at an industrial scale, and with lower environmental impact. The constraints include high costs, problems with health and safety resulting from the use of specific toxic compounds, and solvent waste management^19^.

One of the most cost-effective and conventional technique employed for pre-treatment of cellulose from SCB fibers is alkaline treatment. Sodium hydroxide when treated with fiber produces sodium salt of alcoholic group which helps to dissolve low molecular weight impurities in water. Consequently, the roughness of the cellulose fiber surface increases, and the number of hydrophilic groups decreases. A tiny portion of the lignin, hemicellulose, oils, and waxes that cover the outside of the fiber and expose its short-length, depolymerized cellulose content is also get removed in process. Hence, alkali-treated fibers have a rougher surface, better packing, and structural interlocking which help in improving the properties of polymer composites^12,16,20,21^.

After the successful pre-treatment of cellulose fibers, chemical modifications can be performed on the pretreated cellulosic fibers using different chemical reagents, some of which are listed below:

1. ***Esterification treatment***: The esterification process stabilizes the cell wall, resulting in a variance in plant cellulose fibers and a greater intake of moisture. It also produces a rough fiber surface with low void contents^22–24^.
2. ***Coupling***: Covalent bonds between the fiber and matrix are created because of the treatment with coupling agents containing double bonds. Fiber treatments usually reduce the absorption of water and the loss of physical qualities when the fiber is wet, but only the formation of covalent connections between the fiber and matrix can prevent deterioration at the matrix interface caused by submersion in water and drying^25,26^.
3. ***Silane Treatment***: Two types of reactions are perfromed by common silane coupling materials. The first is the addition of a silane agent in the form of alkoxysilane, which can react with surface hydroxyl groups. The second end connects with the polymer matrix^20,27^.

Compared to the fabrication of ceramic matrix composites, carbon matrix composites, or metal matrix composites, the manufacturing of relatively easier polymer matrix composite materials is always preferred^1^. Many manufacturing processes, including spraying, lay-up, resin transfer molding, compression molding, pressing, and stir casting, can be used to manufacture composite materials^28^.

This paper discusses about the results of esterification reaction using phthalic anhydride and phthaloyl chloride and oxidation reaction performed using sodium percarbonate on pre-treated cellulosic fibers derived from sugarcane bagasse. The fibers were initially treated with 5% NaOH after soaking the raw, untreated, manually cut hard rind of previously hydrolyzed SCB. The results were analyzed using FTIR, XRD, TGA, and SEM to confirm successful modifications, as these methods enhanced the adhesion of the composites to nonpolar matrices.

##  Materials and methods

### 1) Extraction and Processing

**Fig. 1:**
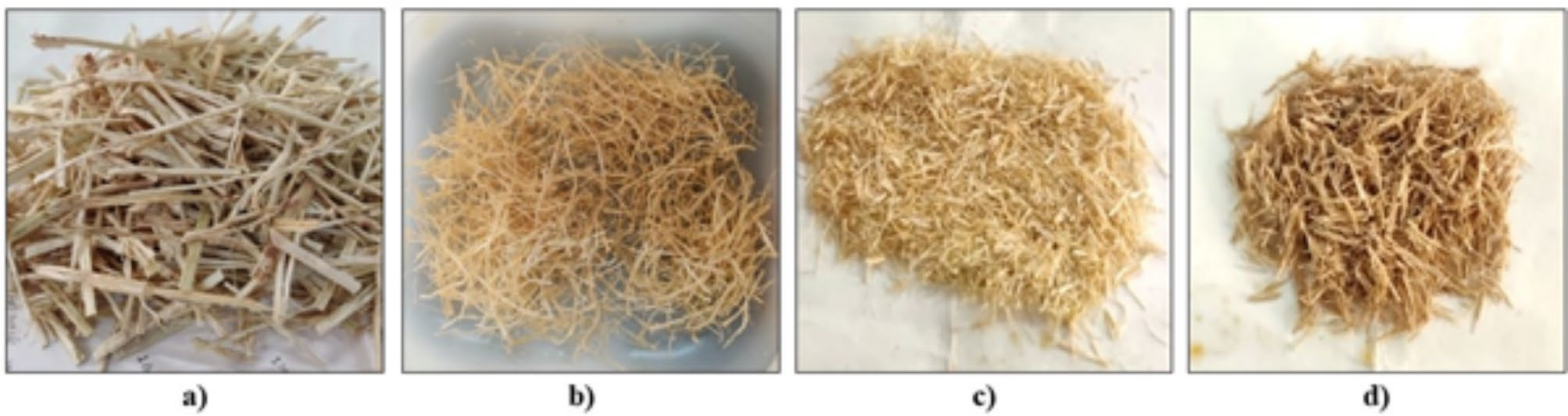
Extraction of cellulose fibers from Sugarcane bagasse and further chemical treatment; (**a**) raw bagasse cut into small pieces, sun-dried, washed with water and oven-dried, (**b**) Cellulose fibers after pre-treatment with alkaline solution, (**c**) Pre-treated cellulose fibers, dried and choppped, (**d**) Esterified cellulose fibers for surface modifications.

 SCB, which is the leftover material from sugarcane juice extraction, is obtained directly from local vendors. The hard outer rind of the bagasse was manually separated from the soft interior pitch. Using scissors, the bagasse was cut into small sections (approximately 4–5 cm), after which any dark portions were removed. To prevent fungal growth, the fibers were thoroughly sun-dried after this process. (Fig. 1a) After soaking in water for 5–6 h to remove sugar residues, the bagasse was oven-dried for 1 h at 60 °C and then air-dried for 24 h to eliminate any odor^29,30^.

### 2) Pre-treatment

Approximately 20–25 g of washed and dried SCB was taken for alkaline treatment. They were immersed in 5% NaOH for 4–5 h, to remove most of the lignin content^31^. Later, the fibers were rinsed with hot, distilled water and neutralized with dilute acetic acid until a neutral pH was obtained by the fibers which was confirmed using litmus paper^32,33^. The fibers were then dried in an oven at 60 °C for about an hour, followed by air-drying for 24 h to remove excess moisture. (Fig. 1b) Finally, the dried fibers were manually chopped and crushed further for chemical treatment, as shown in Fig. 1c.

### 3) Chemical Treatment

#### Esterification of cellulose pre-treated from bagasse fibers using phthalic anhydride

Approximately 2 g of pretreated and crushed SCB fibers was placed in a round bottom flask. A solution containing phthalic anhydride (3.5 g), sodium acetate (8.75 g) as a base, and glacial acetic acid (50 ml) was added to a round-bottom flask (RBF). The reaction mixture was kept at 90 °C for 6 h. The modified fibers were cooled overnight, washed thoroughly with distilled water, and then dried in an oven at 60 °C for an hour before the final analysis, as observed in Fig. 1d^34^.

#### Esterification of cellulose pre-treated from bagasse fibers using phthaloyl chloride

Briefly 2 g of pretreated, crushed bagasse fibers was mixed with phthaloyl chloride (3.5 g) and pyridine solvent (50 ml) in a RBF. The mixture was then refluxed in a steam bath for 3–4 h and allowed to cool. After proper rinsing with distilled water, the fibers were oven-dried at 60 °C for about an hour. The samples were prepared for further analysis^35^.

#### Oxidation of cellulose fibers extracted from sugarcane bagasse using sodium percarbonate

Initially, a catalytic solution was prepared with potassium dichromate (K_2_Cr_2_O_7_) (30 mg) and Adogen (Aliquat 336) (81 mg), a phase transfer catalyst, dissolved in 1,2-dichloroethane (50 ml). The solution was stirred for 30 min at room temperature. Then, 2 g of pretreated bagasse fibers was added, along with sodium percarbonate (3.5 g). The mixture was stirred for 24 h at 80 °C, cooled, and filtered. The treated fibers were rinsed with a little chloroform and distilled water and then dried at room temperature for substitution analysis^36,37^.

## Results and analysis

### FTIR analysis

#### Raw untreated bagasse fibers and pretreated fibers with 5% NaOH solution

**Fig. 2:**
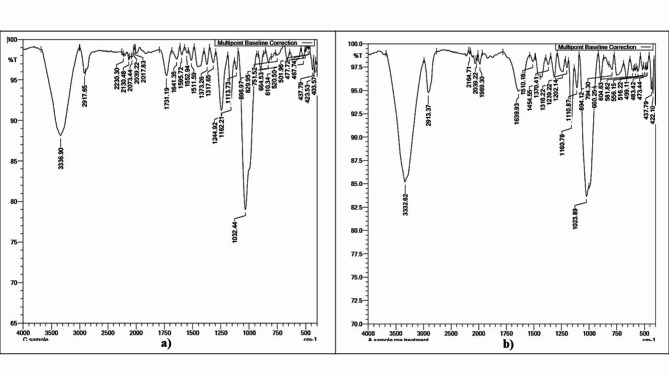
FTIR results; (**a**) Raw sugarcane bagasse, (**b**) 5% NaOH-treated sugarcane bagasse fibers.

From Fig. 2a, the 3100–3600 cm^−1^ peak belongs to the hydrogen-bonded -OH stretching due presence of cellulose as well as hemi-cellulose and lignin present in raw, untreated bagasse. The peak at 2917 cm^−1^ is due to the C-H stretching vibration that can validate hemicellulose^38^. The vibrational stretching of carbonyl groups (C = O) can be attributed to the absorption signal at 1731 cm^−1^. The peak at approximately 1200 cm^−1^ is attributed to the C-O stretching of the acetyl group, which is also found in hemicellulose^39–41^. The vibrational frequency of hemicellulose and lignin C-O can be explained by the stretching at 1032 cm^−1^^42^.

As shown in Fig. 2b, the broad peak at 3100–3600 cm^−1^ is attributed to hydrogen-bonded -OH stretching. The increase in intensity can be explained by the greater number of exposed -OH groups on the cellulose surface. The decrease in peak intensity around 1200 cm^−1^ corresponding to the C-O stretching of the acetyl group found in hemicellulose, proves its partial elimination^43^. The vibrational frequency of hemicellulose and lignin C-O could be explained by the presence of stretch at 1023 cm^−1^ with decrease in its intensity considerably. These findings provide some evidence of a decrease in hemicellulose, wax, and lignin contents after alkaline pretreatment^42,44,45^.

Low-molecular-weight components are dissolved by ionizing the hydroxyl group to the alkoxides. However, higher NaOH concentrations have been linked to excessive fiber delignification, which weakens the fibers. Alkali treatment may also decrease the amount of polymerization, the amount of lignin, and the amount of hemicellulose in the fiber^39,41^.

#### Chemical functionalization of SCB cellulose fibers

**Fig. 3:**
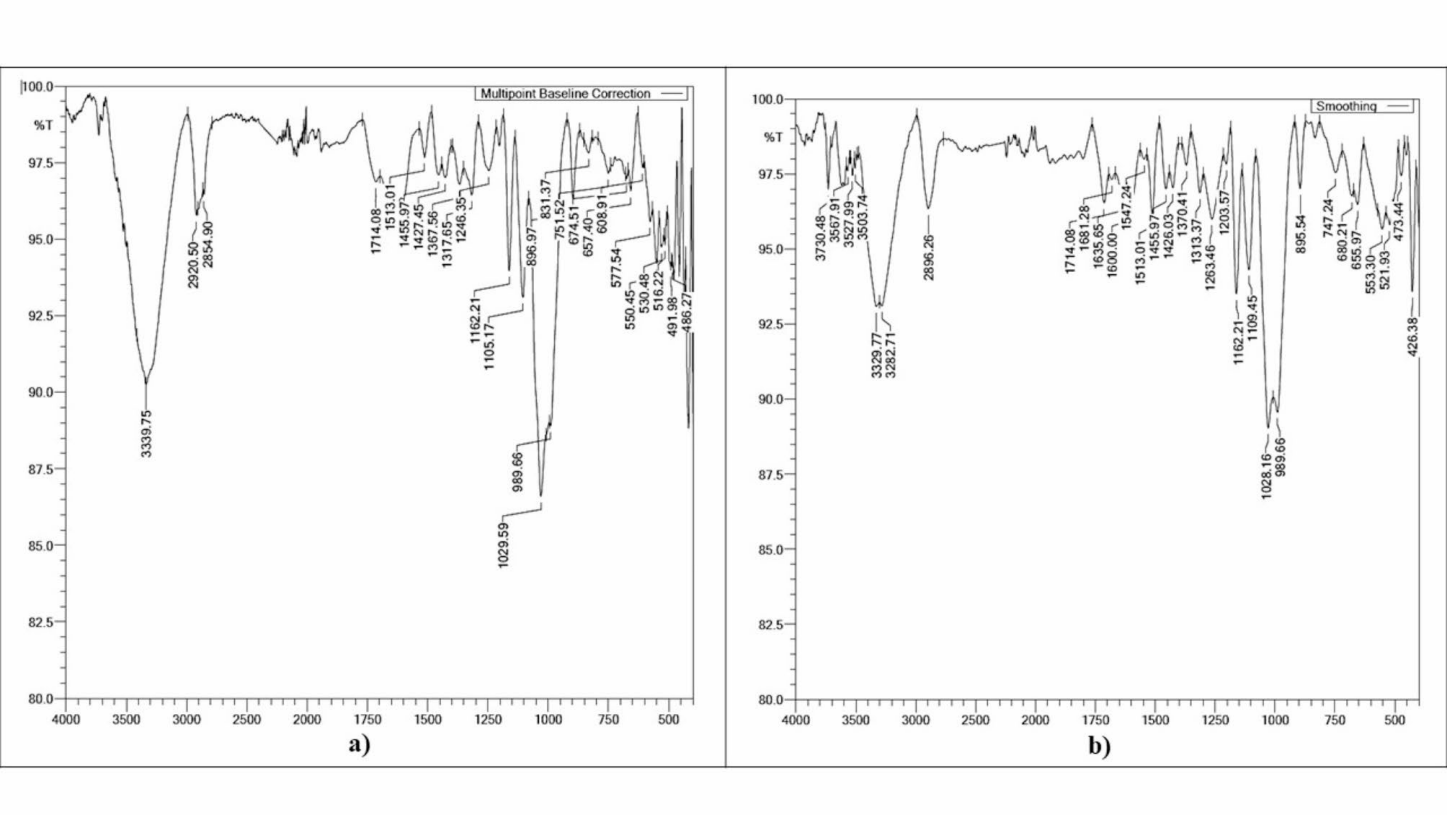
FTIR results; (**a**) phthalic anhydride treated cellulose fibers of SCB, (**b**) phthaloyl chloride treated cellulose fibers of SCB.

As shown in Fig. 3a, after treating cellulose fibers with phthalic anhydride, broad -OH stretching was observed between 3100 and 3500 cm^−1^ with a decrease in intensity. A C-H stretching peak is observed at approximately 2800–2900 cm^−1^^39^. To confirm the substitution of phthalic anhydride as a phthalic ester for cellulosic -OH groups, the following observations were made: a weak peak corresponding to C = O stretching at 1714 cm^−1^, a stretching frequency denoting aromatic/cyclic alkene at 1500–1600 cm^−1^, and aromatic ester signals estimated with peaks around 1200–1300 cm^−1^^46^.

The IR data provides a preliminary analysis of lignin and hemicellulose removal and indicates possible esterification on the cellulose surface. A more conclusive assessment of surface esterification can be made by combined analysis using XRD, SEM, and TGA results.

Figure 3b shows a broad peak at -OH stretching between 3100 and 3500 cm^−1^ with a decrease in intensity corresponding to the phthaloyl chloride treated fibers of the SCB^39^. To confirm the substitution of phthaloyl chloride as a phthalic ester for cellulosic -OH groups, the following observations were made; stretching for C = O ester groups was observed at 1715–1750 cm^−1^ and aromatic esters corresponded to the peaks at 1200–1300 cm^−1^. Aromatic/cyclic alkenes can be identified by the peak at 1500–1600 cm^−1^^46^.

The data above is a preliminary analysis of lignin and hemicellulose removal and suggests esterification on the cellulose surface, but further conclusive determination of surface esterification requires comparison with data from XRD, SEM, and TGA.

**Fig. 4:**
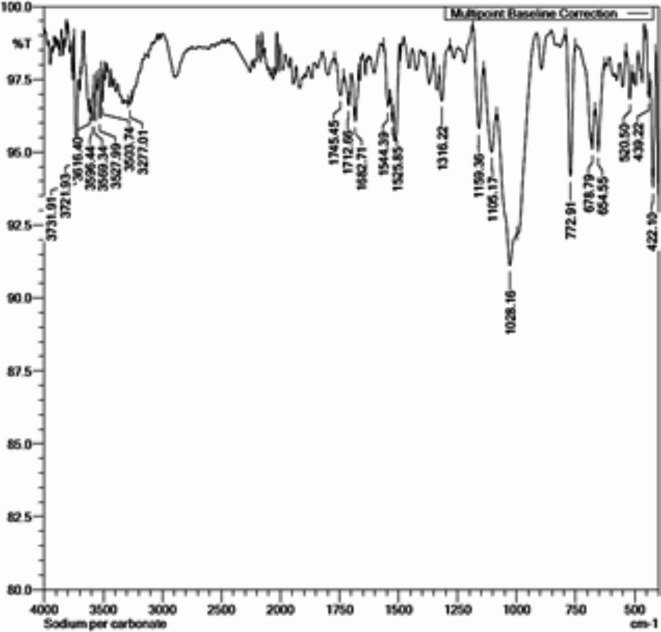
FTIR results of sodium percarbonate treated cellulose fibers of SCB.

When treated with sodium percarbonate for the oxidation of cellulose fibers, the peaks in the ranget 3100 to 3500 cm^−1^ indicate a decreased intensity as shown in Fig. 4. This might be due to relatively lower number of -OH groups on surface which attributable to oxidation reaction. The peak with stretching frequencies of approximately 1690 and 1720 cm^−1^ confirms the presence of carbonyl groups in the fiber and the weak neighbouring peak near 1745 cm^−1^ may denote conjugated ketones which proves the oxidation of the cellulosic -OH groups.

### XRD analysis

To analyze the crystallinity of the chemically treated fibers, X-ray diffraction (XRD) [Rigaku Miniflex 600 (5th gen), CIF, MAHE] was used. The sample was scanned in the 2θ range between 0 and 60°. The crystallinity index was calculated using the following formula,

$$\:Crystallinity\:Index=\:\frac{Area\:of\:crysatlline\:peaks}{Area\:of\:all\:\left(crystalline+amorphous\right)\:peaks}\times\:100\:$$

**Fig. 5:**
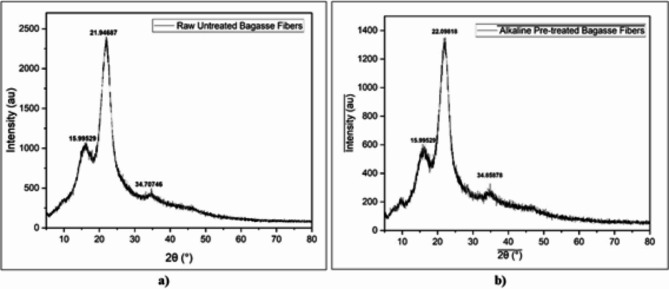
XRD data; (**a**) Raw sugarcane bagasse. (**b**) 5% NaOH-treated sugarcane bagasse fibers.

Table 1 indicates that the crystallinity of the treated fibers decreased compared to that of the raw and pretreated bagasse fibers which proves its significance in removing most of the lignin, hemicellulose, and other impurities. Initially, after alkaline treatment, the number of exposed -OH groups slightly increased while after chemical modification, the number of exposed -OH groups considerably decreased. (Fig. 5)^33,39,41^.

**Table 1:** Crystallinity indices of untreated, NaOH pretreated, and chemically modified cellulose fibers of SCB.

Sr. No.	Sample	Crystallinity Index (I_C_) %
1.	Raw untreated bagasse	59%
2.	Pretreated bagasse fibers	62%
3.	Phthalic anhydride-treated fibers	51%
4.	Phthaloyl chloride-treated fibers	54.4%
5.	Sodium percarbonate-treated fibers	48.5%

**Fig. 6:**
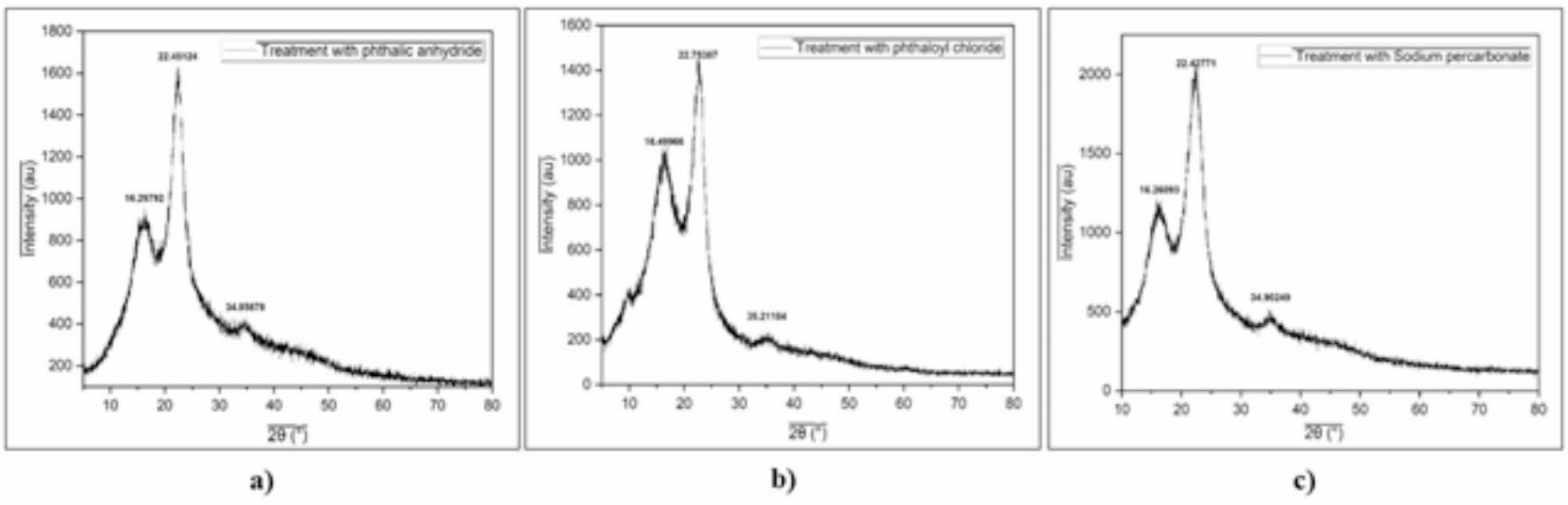
XRD data of chemically modified cellulose fibers of SCB; (**a**) phthalic anhydride treated cellulose fibers of SCB, (**b**) phthaloyl chloride treated cellulose fibres, (**c**) sodium percarbonate treated cellulose fibers.

A significant decrease in the crystallinity index was observed after chemical treatment with phthalic anhydride (Fig. 6a), phthaloyl chloride (Fig. 6b), and sodium percarbonate (Fig. 6c), as the -OH groups were successfully esterified and oxidized thus reducing the crystallinity and increasing the degree to which the materials were amorphous enough to bind well with the polymer matrix^47^.

### SEM analysis

Scanning electron microscopy (SEM) was used to analyze the morphology of the raw and chemically treated bagasse fibers. The sample was initially sputtered, and SEM (ZEISS EVO 18) analysis was carried out under vacuum on an aluminium stub.

**Fig. 7:**
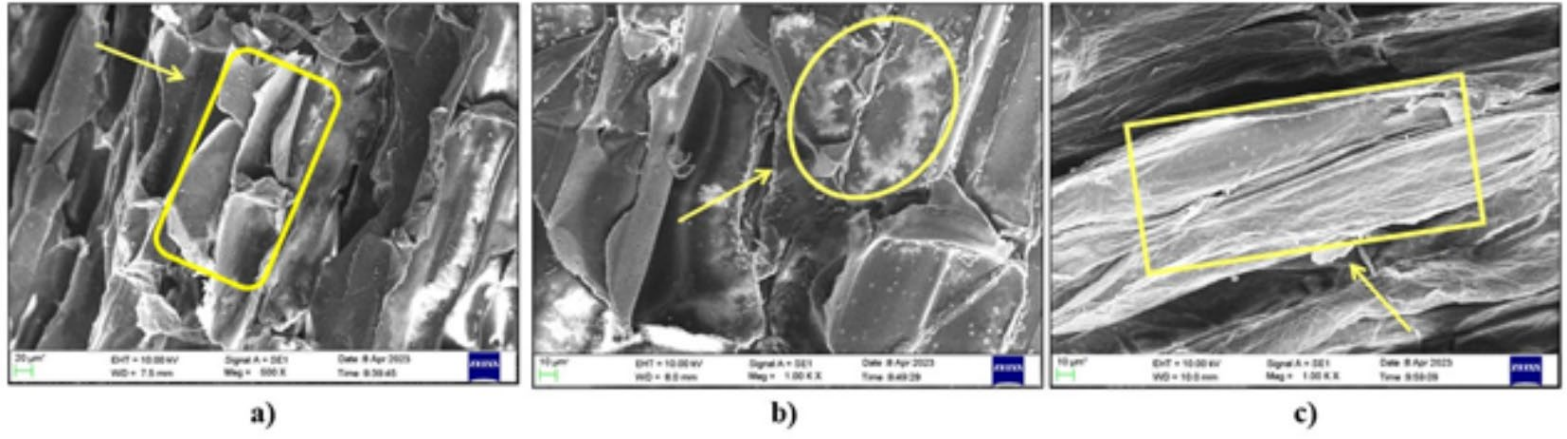
SEM images; (**a**) Raw, untreated sugarcane bagasse, (**b**) & (**c**) 5% NaOH-treated sugarcane bagasse fibers.

The SEM morphology of the untreated fibers at various magnifications revealed the presence of significant amounts of extractives, waxes, lignin, and hemicellulose, which appeared as white layers, and exhibited a nail-like morphology with tiny discontinuous sections that served as continuous fiber covering layers for cellulosic material, as shown in Fig. 7a and b. These materials exhibit a weak affinity for hydrophobic resin systems^38,40^.

SEM provides encouraging evidence for the removal of extractives, which reveals the smooth accessible surface of the cellulose fibers, and alkaline-treated fibers demonstrate the breakdown of lignin. The matte surface shown in SEM Fig. 7c indicates the semi-crystalline nature of the material^39,40,44,45^.

The lignin breaks down in the chemically treated fibers, and SEM provides encouraging evidence for the elimination of extractives and cellulose, which endows the fibres with roughness. A surface modified with these chemicals make it easier for the fiber and matrix to bind at the interface. The surface of these modified fibers has increased hardness, and microfibril breakage is also apparent^6,33^.

**Fig. 8:**
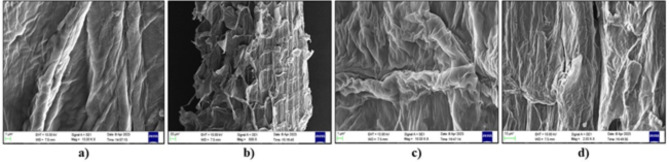
SEM images of chemically modified cellulose fibers of SCB; (**a**) phthalic anhydride treated cellulose fibers of SCB, (**b**) phthaloyl chloride treated cellulose fibres, (**c**) and (**d**) sodium percarbonate treated cellulose fibers.

Chemically altered fibers exhibit increased roughness (Fig. 8b and d) compared to the glossy surface of pretreated fibres, which ultimately improves the adhesion between the fibers and the polymer matrix^47^. The fiber bundles that formed can also be observed in Fig. 8a and c, which signify uniform treatment of the fiber surface. Both esterification and oxidation gave satisfactory results according to the SEM images.

### TGA analysis

For the analysis of thermal stability, TGA (Model TG/DTA 200) was employed. A sample weighing 2 mg was placed in a platinum pan and tests were performed using a programmed temperature of 10 °C/min in a temperature range from room temperature (25 °C) to 800 °C in an inert N_2_ atmosphere.

Due to the loss of volatile substances, the weight loss between 50 °C and 70 °C is between 2% and 4%. Hemicellulose and cellulose begin to gradually degrade at temperatures greater than 230 °C; more specifically, between 345 °C and 365 °C, cellulose appears to degrade and hemicellulose begins to degrade. Lignin is responsible for decomposition temperatures above 400 °C. Up to 230–250 °C, the maximum processable temperature at the minimal weight loss, and thermal stability can be observed^39–41,45^.

Dehydration and decarboxylation, which include breaking of the C-C, C-H, and C-O bonds, occur at temperatures above 230 °C, consistent with the thermal properties already stated for many natural fibers. Complete decomposition of the material occurs at approximately 475–500 °C^39,48^.

Table 2 below provides information about T_20%_ which represents the decomposition temperature at which 20% weight loss occurs. It was chosen because every sample lost volatile substances and trapped moisture around this temperature and the data remained almost consistent for every fiber analysis. Then, we have T_max_ which is the temperature at which maximum decomposition has occurred.

**Table 2:** Thermal stability of raw, NaOH pretreated, and chemically modified cellulose fibers of SCB.

Sr. No.	Sample	T_20%_	T_max_
1.	Raw untreated Bagasse	212.6 °C	419 °C
2.	NaOH pretreated Bagasse fibers	245 °C	446 °C
3.	Phthalic anhydride-treated fibers	222.4 °C	460 °C
4.	Phthaloyl chloride-treated fibers	191.5 °C	496 °C
5.	Sodium percarbonate-treated fibers	239 °C	459 °C

**Fig. 9:**
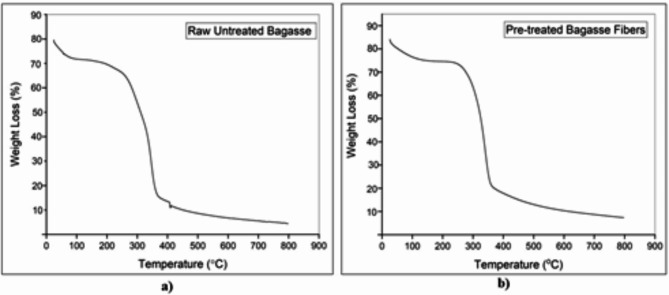
TGA results; (**a**) Raw, untreated sugarcane bagasse, (**b**) 5% NaOH-treated sugarcane bagasse fibers.

The above observations showed an increase in the T_max_ decomposition temperature for the pretreated fibers compared with that for the raw and untreated bagasse fibers which proves the increase in thermal stability of the former. (Figure 9a and b)

**Fig. 10:**
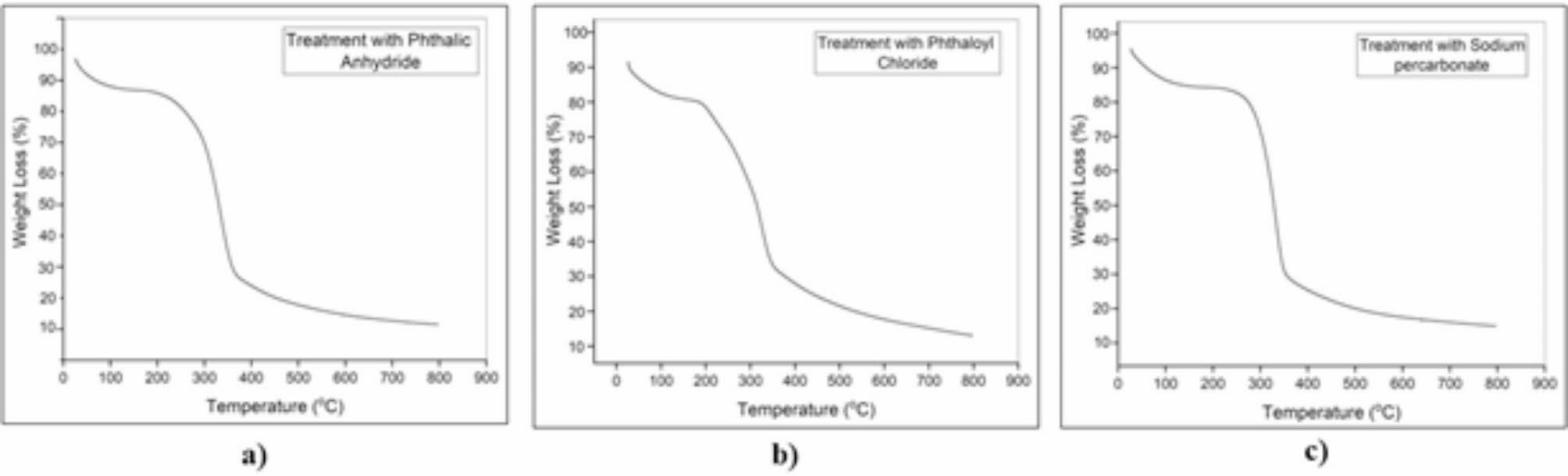
TGA graph of chemically modified cellulose fibers of sugarcane bagasse fibers; (**a**) phthalic anhydride treated cellulose fibers of SCB, (**b**) phthaloyl chloride treated cellulose fibres, (**c**) sodium percarbonate treated cellulose fibers.

- To further understand the thermal stability of these materials, other chemical treatments such as esterification with phthalic anhydride (Fig. 10a), phthaloyl chloride (Fig. 10b), and oxidation with sodium percarbonate (Fig. 10c) were tested against pretreated bagasse fibres. They showed similar results, with the T_max_ decomposition temperature increasing after treatment which can be taken to prove the increased thermal resistance characteristic of the modified fibres^39,44^.

## Conclusions

Sugarcane bagasse fibers show promising potential for use in polymer composite applications, offering a sustainable and eco-friendly alternative to conventional materials. Pretreatment of raw bagasse after hydrolysis using 5% NaOH solution to obtain cellulose and eliminate most of the hemicellulose, lignin, wax, etc., was successful when we compared the results with existing data. Investigating the physicochemical characteristics of chemically functionalized bagasse fibers, such as their morphology, chemical composition, thermal stability, and crystallinity index, yielded the following significant findings:

- Phthalic anhydride treatment with sodium acetate as a base at a 1:2 ratio for esterification decreased the crystallinity as determined via XRD; moreover, an increase in the thermal stability (T_max_=460 °C) and in the morphology of the fiber bundles observed via SEM supported these findings. The FTIR spectrum also provides preliminary evidence of substituted esters in the cellulose chain.
- Similarly, treatment of pretreated bagasse fiber with phthaloyl chloride provided a preliminary evidence of ester modifications to the cellulosic -OH groups according to the FTIR data, while XRD confirmed a reduction in the crystallinity (54.4%), TGA showed a significant increase in thermal stability (T_max_=496 °C) and SEM analysis revealed a rough surface with visible fiber bundles confirming the credibility of the treatment.
- For a greener approach, oxidation was performed using sodium percarbonate and a phase transfer catalyst (Adogen) to test its performance in terms of cellulose modification. The FTIR results provided evidence for oxidation of -OH. An increase in the thermal stability of the fibers (T_max_=459 °C), a decrease in the crystallinity index (48.5%), and a hardened surface morphology were proven by TGA, XRD and SEM data, respectively.

Thus, compared to those of raw bagasse fibers, the use of untreated bagasse fibers, essential chemical treatments, and pretreatment steps can be used to modify cellulose specifically for composite applications. Our results demonstrate the removal of lignin and hemicellulose, as well as surface modification of the cellulose fibers. Further studies are required to evaluate the performance of modified fibers on specific composite material application which depend on type of polymer used, method of preparation and fabrication techniques employed and other parameters.

## Future scope

Natural fiber-based composites provide eco-friendly alternatives for products such as cars, aerospace components, ceiling panels, and packaging. Despite growing deforestation, the demand for wood products is increasing. Green technology is concentrating on creating wood substitutes by combining wood materials with polymers to produce affordable, high-performance, and long-lasting products. Sugarcane bagasse, which is easily accessible as a significant agricultural/food waste, has emerged as an excellent alternative. Chemical modification can be performed to meet specific needs for various composite applications.

## Data Availability

The required data has been presented and made available within the manuscript.
